# Smoking is associated with pneumonia development in lung cancer patients

**DOI:** 10.1186/s12890-020-1160-8

**Published:** 2020-05-01

**Authors:** Jung Won Heo, Chang Dong Yeo, Chan Kwon Park, Sung Kyoung Kim, Ju Sang Kim, Jin Woo Kim, Seung Joon Kim, Sang Haak Lee, Hye Seon Kang

**Affiliations:** 1 Division of Pulmonary, Critical Care and Sleep Medicine, Department of Internal Medicine, Eunpyeong St. Mary’s Hospital, College of Medicine, The Catholic University of Korea, Seoul, Republic of Korea; 2 Division of Pulmonary, Critical Care and Allergy, Department of Internal Medicine, Yeouido St. Mary’s Hospital, College of Medicine, The Catholic University of Korea, Seoul, Republic of Korea; 3 Division of Pulmonary, Critical Care and Allergy, Department of Internal Medicine, St. Vincent’s Hospital, College of Medicine, The Catholic University of Korea, Seoul, Republic of Korea; 4 Division of Pulmonary, Critical Care and Sleep Allergy, Department of Internal Medicine, Incheon St. Mary’s Hospital, College of Medicine, The Catholic University of Korea, Seoul, Republic of Korea; 5 Division of Pulmonary, Critical Care and Sleep Medicine, Department of Internal Medicine, Uijeongbu St. Mary’s Hospital, College of Medicine, The Catholic University of Korea, Seoul, Republic of Korea; 6 Division of Pulmonary, Critical Care and Allergy, Department of Internal Medicine, Seoul St. Mary’s Hospital, College of Medicine, The Catholic University of Korea, Seoul, Republic of Korea; 7 Cancer Research Institute, College of Medicine, The Catholic University of Korea, Seoul, Republic of Korea; 8 Division of Pulmonary, Critical Care and Allergy, Department of Internal Medicine, Bucheon St. Mary’s Hospital, College of Medicine, The Catholic University of Korea, 327, Sosa-ro, Bucheon-si, Gyeonggi-do 14647 Republic of Korea

**Keywords:** Pneumonia, Smoking, Lung cancer

## Abstract

**Background:**

Various host factors can promote pneumonia susceptibility of lung cancer patients. However, data about risk factors for pneumonia in lung cancer patients receiving active treatments such as chemotherapy, radiotherapy, and surgical intervention are limited. Thus, the purpose of this study was to identify risk factors for pneumonia development in lung cancer patients.

**Methods:**

The present study used a lung cancer cohort of the Catholic Medical Center at the Catholic University of Korea from January 2015 to December 2018. Pneumonia was defined by the presence of a new or progressive infiltration on chest imaging together with any of the following: new onset purulent sputum, change in character of chronic sputum, and fever. We ruled out noninfectious infiltration such as drug or radiation toxicity and hydrostatic pulmonary edema. We especially excluded those if computed tomography revealed sharp demarcation consolidation or ground glass opacity limited radiation field.

**Results:**

A total of 413 patients were enrolled in this study. Pneumonia occurred in 118 (28.6%) patients. The pneumonia group had significantly worse overall survival (OS) than the *non*-pneumonia group (456.7 ± 35.0 days vs. 813.4 ± 36.1 days, log rank *p* < 0.001). In patients with pneumonia, OS was shorter in ex-smokers and current smokers than in never smokers (592.0 ± 101.0 days vs. 737.0 ± 102.8 days vs. 1357.0 days, log rank *p* < 0.001). Age (hazard ratio [HR]: 1.046; 95% confidence interval [CI]: 1.019–1.074; *p* = 0.001), clinical stage IV (HR: 1.759; 95% CI: 1.004–3.083; *p* = 0.048), neutropenia (HR: 2.620; 95% CI: 1.562–4.396; *p* < 0.001], and smoking (HR: 2.040; 95% CI: 1.100–3.784; *p* = 0.024) were independent risk factors of pneumonia development in lung cancer patients in multivariate analysis. In subgroup analysis for patients treated with chemotherapy, age (HR: 1.043; 95% CI: 1.012–1.074; *p* = 0.006), neutropenia (HR: 3.199; 95% CI: 1.826–5.605; *p* < 0.001), and smoking (HR: 2.125; 95% CI: 1.071–4.216; *p* = 0.031) were independent risk factors of pneumonia development.

**Conclusions:**

Smoking and neutropenia were risk factors affecting pneumonia development in the total group and subgroup of patients with lung cancer.

## Background

Lung cancer is the most common cause of cancer-related deaths worldwide [1]. The impact of pneumonia on cancer population is uniquely severe, accounting for more morbidity and mortality than other infectious complications [2]. Both cancer and cancer related treatments can alter immune responses of hosts. Chemotherapy-induced cytopenia, bone marrow infiltration by tumor, impaired pathogen detection, and dysregulated inflammation are host factors that can promote bacterial pneumonia susceptibility in cancer patients. Coexisting structural lung diseases such as emphysema and bronchiectasis can also affect the occurrence of pneumonia in cancer patients [3]. Further, cancer patients are more likely to be exposed to resistant bacteria because of their frequent hospital visits.

Clinical studies have identified several risk factors for postoperative pneumonia in lung cancer patients. Well-known risk factors are as follows: old age (> 60 years), histopathological type of squamous cell carcinoma [4], smoking (current or previous), and the extent of excision of more than one lobe [5]. Risk factors for post bronchoscopy pneumonia in lung cancer patients are older age (> 70 years), current smoking, and central location of the tumor [6]. Previous studies have focused on pneumonia occurrence after surgery or bronchoscopy in lung cancer patients. However, data about risk factors for pneumonia in lung cancer patients receiving palliative care or active treatment including surgical intervention, chemotherapy, radiotherapy, or tyrosine kinase inhibitor (TKI) are limited. Thus, the objective of this study was to compare clinical factors between pneumonia and non-pneumonia groups and identify risk factors of pneumonia occurrence in lung cancer patients.

## Methods

### Data source

Seven medical centers at the Catholic University of Korea have consecutively enrolled patients with lung cancer since October 2014. The Catholic Medical Center (CMC) lung cancer registry consists of seven multi-centers (Seoul St. Mary’s Hospital, Yeouido St. Mary’s Hospital, Eunpyeong St. Mary’s Hospital, Uijeongbu St. Mary’s Hospital, Bucheon St. Mary’s Hospital, Incheon St. Mary’s Hospital, and St. Vincent’s Hospital) in the capital region of South Korea. Our lung cancer cohort enrolled lung cancer patients confirmed by tissue biopsy. We analyzed data of patients from Seoul St. Mary’s Hospital and Bucheon St Mary’s Hospital among cohort hospitals because their medical charts related to the development of pneumonia were available for review. CMC lung cancer registry collected smoking history including smoking status, pack-year, and duration of smoking cessation. If the patient continued smoking until diagnosis or stopped smoking within 1 month of being diagnosed with lung cancer, he or she was defined as a current smoker. Ex-smoker was defined as the patient who quitted smoking at least 1 month before the diagnosis of lung cancer. The patient who had smoked fewer than 100 cigarettes in their lifetime or had never smoked as never smoker [7]. Lung function test was performed according to the American Thoracic Society/European Respiratory Society standardization guidelines. Clinical staging was performed using the 7th edition of TNM staging system until 2017, and that for lung cancer patients diagnosed since 2018 was based on the 8th edition of TNM staging, which was authorized by the American Joint Committee on Cancer on January 1, 2018. Patients in the first-line of therapy are those who have received one curative or palliative line of treatment. Second-line (third-line) therapy was defined as the treatment after discontinuing first-line (second-line) therapy, either for intolerance or for progressive/recurrent. In contrast, patients in the 0 line of therapy group did not receive any therapy. Clinical data including stage, pathology, treatment modality, and survival were systematically recorded by qualified data managers to improve the accuracy of data. This study was approved by the Clinical Research Ethics Committee of the Catholic Medical Center (approval number: XC140IMI0070).

### Pulmonary infection diagnosis criteria

Pneumonia was defined by the presence of a new or progressive infiltration on chest radiography or computed tomography (CT), together with any of the following: purulent sputum and fever [8]. However, differential diagnosis of pulmonary infiltration is expansive, including non-infectious mimics such as drug injury, radiation toxicity, and hydrostatic pulmonary edema [8–10]. If bacterial pneumonia is suspected, microbiological diagnosis, such as bronchoalveolar lavage or bronchial aspiration, was performed to differentiate it from radiation and cytostatic pneumonitis. However, these methods were invasive, so it was impossible to perform procedures in all patients. And the sensitivity has varied widely, ranging from 30 to 80% [11, 12]. In patients undergoing antibiotics treatment, the sensitivity is much lower [13]. So, positive blood cultures, isolation of pathogen from sputum, transtracheal aspirate, and bronchial washing were not compulsory inclusion criteria. Especially, we excluded those when computed tomography revealed relatively sharp demarcation consolidation or ground glass opacity (GGO) with limited radiation field suggesting radiation pneumonitis. We also identified procalcitonin level. Procalcitonin is a useful marker to distinguish bacterial pneumonia from radiation pneumonitis in solid tumor patients [14, 15]. In addition, bacterial pneumonia was diagnosed by confirming radiologic and clinical improvement after empirical antibiotics.

### Data collection

Data of basic demographics were collected, including age, gender, body mass index (BMI), smoking (current or previous smoker vs. never smoker), comorbid condition, pathologic finding (adenocarcinoma vs. squamous cell carcinoma vs. small cell lung cancer [SCLC] vs. adenosquamous cell carcinoma vs. large cell carcinoma vs. mesothelioma vs. others), line of therapy, neutropenia, pulmonary function test, the presence of endobronchial lesion in bronchoscopic finding, clinical stage (I–IV), and treatment modality (no therapy vs. surgery vs. chemotherapy vs. TKI vs. concurrent chemoradiation therapy [CCRT] vs. stereotactic body radiation therapy [SBRT] vs. radiation therapy vs. neo-adjuvant CCRT).

### Statistical analysis

The Pearson’s Chi-square tests and Student’s *t*-test were used to compare clinical characteristics between lung cancer patients with and those without pneumonia. The survival curve according to the pneumonia development was analyzed using the Kaplan-Meier method. Hazard ratios (HRs) and corresponding 95% confidence intervals (Cis) were calculated for predictos that were significant in multivariate Cox regression analysis. A two-sided *P* value < 0.05 was considered to be statistically significant. All statistical analyses were performed using SPSS for Windows software (ver. 20.0; IBM Corp., Armonk, NY, USA).

## Results

A total of 413 patients diagnosed with lung cancer were included. Of these patients, 118 (28.6%) patients developed pneumonia. Of these, 94 patients underwent CT and microorganism were detected in 42 patients. We compared clinical factors between pneumonia and non-pneumonia groups and explored clinical factors predicting pneumonia development. Mean follow-up time was 441.40 ± 326.76 days. Baseline characteristics of these two groups are summarized in Table 1.

**Table 1:** Comparison of baseline characteristics of lung cancer patients with or without pneumonia

Characteristic	Pneumonia (*n* = 118)	Non-pneumonia (*n* = 295)	*p*-value
Age, year	69.79 ± 8.35	66.37 ± 10.60	0.002
Sex, male	98 (83.1)	205 (69.5)	0.005
BMI, kg/m^2^	22.80 ± 2.943	23.52 ± 3.121	0.887
Smoking history			0.002
Never	17 (14.4)	91 (30.8)	0.001
Former	53 (44.9)	113 (38.3)	0.216
Current	48 (40.7)	91 (30.8)	0.056
Pack-years	39.85 ± 23.83	39.89 ± 25.50	0.992
Underlying disease	97 (82.2)	235 (78.9)	0.444
Accompanied cancer	15 (12.7)	47 (75.8)	0.430
Pathology			0.004
Adenocarcinoma	53 (44.9)	183 (62.0)	0.002
SqCC	32 (27.1)	64 (21.7)	0.238
SCLC	29 (24.6)	29 (9.8)	< 0.001
Adenosquamous	1 (0.8)	5 (1.7)	0.516
Large cell	1 (0.8)	2 (0.7)	0.885
NOS	0 (0)	1 (0.3)	0.527
Mesothelioma	0 (0)	0 (0)	
Others	2 (1.7)	8 (2.7)	0.354
Line of therapy			0.067
0	7 (5.9)	24 (8.2)	
1	57 (48.3)	139 (47.3)	
2	25 (21.2)	87 (29.6)	
≥ 3	29 (24.6)	44 (15.0)	
Neutropenia	51 (43.2)	56 (18.9)	< 0.001
PFT			
FVC, % pred	82.25 ± 16.810	87.77 ± 19.349	0.218
FEV_1_, % pred	78.39 ± 19.219	83.93 ± 22.878	0.083
FEV_1_/FVC < 70%	47 (49.5)	93 (40.8)	0.151
FEV_1_/FVC	0.90 ± 0.297	0.93 ± 0.259	0.103
Clinical stage			< 0.001
I	15 (12.7)	100 (33.9)	< 0.001
II	10 (8.5)	19 (4.6)	0.476
III	47 (39.8)	50 (16.9)	< 0.001
IV	46 (39.0)	124 (42.0)	0.534
Treatment modality			< 0.001
No therapy	7 (5.9)	22 (7.5)	0.567
Surgery	26 (22.0)	107 (36.6)	0.004
Chemotherapy	47 (39.8)	75 (25.7)	0.005
TKI	7 (5.9)	47 (16.1)	0.006
CCRT	17 (14.4)	18 (6.2)	0.007
SBRT	0 (0)	2 (0.7)	0.367
RT	11 (9.3)	14 (4.8)	0.083
Neo-adjuvant CCRT	0 (0)	3 (1.0)	0.247

Data are expressed as mean ± standard deviation or number (%) except where otherwise indicated

Abbreviations: BMI, body mass index; SqCC, squamous cell carcinoma; SCLC, small cell lung cancer; NOS, not otherwise specified; PFT, pulmonary function test; FVC, forced vital capacity; pred, predictive value; FEV_1_, forced expiratory volume in 1 s; TKI, tyrosine kinase inhibitor; CCRT, concurrent chemoradiation therapy; SBRT, stereotactic body radiation therapy; RT, radiation therapy

In the pneumonia group, the mean age (69.79 ± 8.35 vs. 66.37 ± 10.60, *p* = 0.002) and the proportion of male were higher (83.1% vs. 69.5%, *p* = 0.005), while the proportion of never smokers (14.4% vs. 30.8%, *p* = 0.001) was lower. Regarding pathological types, SCLC had higher proportion (24.6% vs. 9.8%, *p* < 0.001) while adenocarcinoma had lower proportion (44.9% vs. 62.0%, *p* = 0.002) in the pneumonia group. Neutropenia (43.2% vs. 18.9%, *p* < 0.001) was more frequently accompanied in the pneumonia group. The proportion of clinical stage III (39.8% vs. 16.9%, *p* < 0.001) was higher while that of clinical stage I was lower in the pneumonia group. Regarding treatment modality, chemotherapy (39.8% vs. 25.7%) and CCRT (14.4% vs. 6.2%) had higher proportions whereas surgery (22.0% vs. 36.6%) and TKI (5.9% vs. 16.1%) had lower proportions in the pneumonia group (Table 1). The incidence of pneumonia occurred more frequently during treatment (55%) or after treatment (29.7%) than that during conservative care (15.3%, *p* < 0.001).

**Fig. 1:**
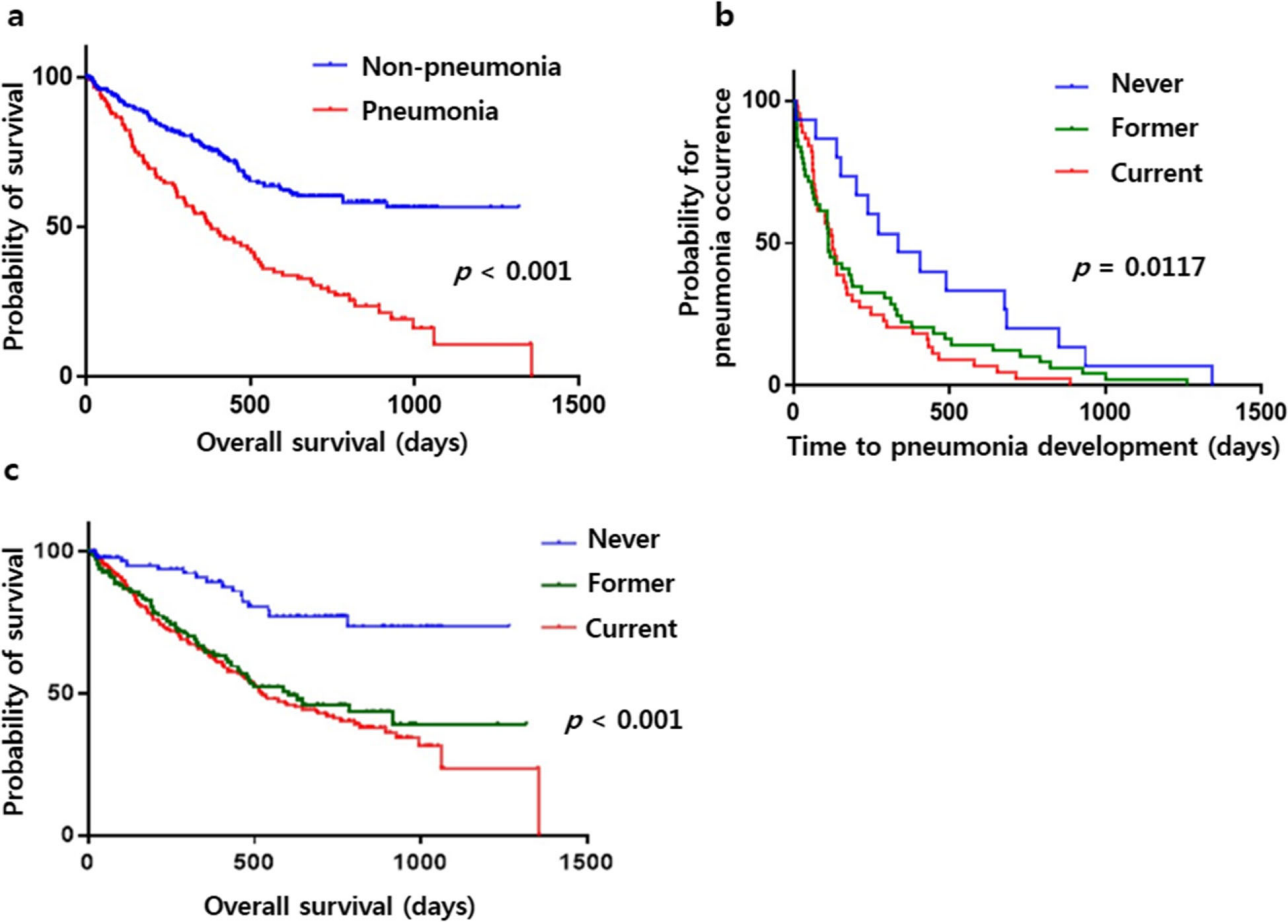
Comparison of survival probability according to occurrence of pneumonia (**a**), probability for pneumonia occurrence according to smoking history (**b**), and survival probability according to smoking history (**c**)

Patients in the pneumonia group showed a shorter median overall survival (OS) than those in the non-pneumonia group (456.7 ± 35.0 days vs. 813.4 ± 36.1 days, log rank *p* < 0.001) (Fig. 1a). Overall survival was calculated based on the date of cancer diagnosis. The total number of patients who died was 175. Of these, 93 patients did not die at our hospital, so the cause of death was unknown. In addition, 36 patients died due to cancer-specific cause, the cancer-specific survival was 1196.2 ± 53.9 days in the pneumonia group and 1178.9 ± 25.1 days in non-pneumonia group (Log rank = 0.866), with no difference between the two groups. We estimated the cumulative incidence of pneumonia cases with exposure to smoking. Those with exposure to smoking showed a higher risk of pneumonia than those without such exposure (i.e., non-smoking) (log rank *p* = 0.0117) (Fig. 1b). Patients with smoking exposure (ex-smoker or current smoker) displayed a shorter median OS than those without exposure to smoking (never smoker group) (592.0 ± 101.0 days vs. 737.0 ± 102.8 days vs. 1357.0 days, log rank *p* < 0.001), although the difference in OS between ex-smokers and current smokers was not statistically significant (Fig. 1c).

Cox proportional hazards model revealed a 2.04-fold increased risk of pneumonia for smokers compared to non-smokers. In addition, older age (HR: 1.046; 95% CI: 1.019–1.074; *p* = 0.001), advanced stage (IV) (HR: 1.759; 95% CI: 1.004–3.083; *p* = 0.048), and neutropenia (HR: 2.620; 95% CI: 1.562–4.396; *p* < 0.001) were found to be independent risk factors for pneumonia development in lung cancer patients (Table 2).

**Table 2:** Cox multivariate proportional hazard analysis for pneumonia development in lung cancer patients

Variable	Hazard ratio	95% Confidence interval	*p*-value
Age	1.046	1.019–1.074	0.001
Clinical staging			0.013
I			
II	0.480	0.246–0.933	0.030
III	1.226	0.512–2.937	0.647
IV	1.759	1.004–3.083	0.048
Neutropenia	2.620	1.562–4.396	< 0.001
Smoking	2.040	1.100–3.784	0.024

We performed subgroup analysis for those who received treatment that included cytotoxic chemotherapy (CCRT, chemotherapy and neoadjuvant CCRT). It has been reported that they show increased risk of developing neutropenia and potentially life-threatening infection due to myelotoxic effects of chemotherapy [16]. In subgroup analysis, 285 patients were treated with therapy that included cytotoxic chemotherapy. Among them, 122 (42.8%) patients received chemotherapy alone, 35 (12.3%) patients received definitive CCRT, 6 (2.1%) patients received neoadjuvant CCRT, and 3 (1.1%) patients received neoadjuvant chemotherapy. Ninety-four (33.0%) and 191 (67.0%) patients were classified into pneumonia and non-pneumonia groups, respectively. Multivariate analysis revealed that neutropenia (HR: 3.199; 95% CI: 1.826–5.605; *p* < 0.001) and smoking (HR: 2.125; 95% CI: 1.071–4.216; *p* = 0.031) were independent risk factors for pneumonia development in lung cancer patients receiving treatment that included cytotoxic chemotherapy (Table 3).

**Table 3:** Cox multivariate proportional hazard analysis for pneumonia development in lung cancer patients who received treatment including cytotoxic chemotherapy

	Hazard ratio	95% Confidence interval	*p*-value
Age	1.043	1.012–1.074	0.006
Line of therapy			0.015
1			
2	0.405	0.212–0.775	0.006
≥ 3	0.904	0.463–1.763	0.767
Neutropenia	3.199	1.826–5.605	< 0.001
Smoking	2.125	1.071–4.216	0.031

In sputum or blood culture, microorganisms were detected in 42 (35.6%) of 118 pneumonia patients. Microbes were detected in 22 (18.6%) patients in the blood culture test and 20 (16.9%) patients in the sputum culture test. The most commonly identified bacterial species was *Staphylococcus aureus* (*n* = 13; 31%). Among them, methicillin-resistant *Staphylococcus aureus* was identified 9 times (21.4%). The second most commonly identified bacterial species was *Klebsiella pneumoniae*.

## Discussion

Lung cancer is a major cause of cancer-related death worldwide [1]. In immunocompromised patients, infections and pneumonia are closely related to a poor prognosis [17, 18]. Therefore, it is important to recognize risk factors associated with pneumonia and prepare prevention strategies to reduce the occurrence of pneumonia in patients with lung cancer.

In our study, patients in the pneumonia group showed a shorter median OS than those in the non-pneumonia group. We explored clinical factors for pneumonia development in lung cancer patients. According to our research, the incidence of pneumonia was 28.6% in the lung cancer population and 33.6% in the subgroup of subjects who received therapy that included cytotoxic chemotherapy. Older age, advanced stage (IV), neutropenia, and smoking were independent risk factors for pneumonia development in lung cancer patients. Age, neutropenia, and smoking were independently associated with pneumonia development in lung cancer patients who received treatment that included cytotoxic chemotherapy.

There has been some research on risk factors for developing pneumonia in patients with lung cancer. Wang et al. [4] have demonstrated that being old aged (> 60 years) and having squamous cell carcinoma histopathological type might be important risk factors of postoperative pneumonia in lung cancer patients after surgery. Liu et al. [5] have reported that patients with older age, smoking, and extent of excision of more than one lobe have a higher risk for pneumonia after lung cancer surgery. Takiguchi et al. [6] have noted that the risk factors for pneumonia after bronchoscopy are old age (≥ 70 years), current smoking, and central location of the tumor. These studies were focused on pneumonia development after surgery or bronchoscopy. However, data about risk factors for pneumonia in lung cancer patients who have received active treatment such as radiation therapy and chemotherapy are limited.

Old age has been associated with pulmonary complication in many studies. Lee et al. [19] have reported that old age is associated with the development of bacterial pneumonia in patients after cytotoxic chemotherapy in advanced lung cancer patients. Age ≥ 64 years was an independent predictors for post-operative pneumonia in lung cancer patients [5]. The elderly have several risk factors of pneumonia occurrences because of their impaired physical conditions. Dysfunction of the immune system is one important cause of pneumonia. Aspirated oropharyngeal secretions into the lung can induce aspiration pneumonia due to swallowing difficulties in old patients [20].

Among risk factors for cancer treatment related pneumonia, neutropenia is one of the most well-known factors. Neutrophils are sensitive to alkylating agents and nucleoside analogs. Absolute neutrophil count decreases with increasing dose of cytotoxic chemoagents [8]. Severe neutropenia defined as a count ≤500/μL is associated with increased severe lung infections caused by bacterial and fungus [21]. Neutropenic pneumonia is affected by rapid onset, duration, severity, and underlying physiologic process [22–24]. Lung cancer is a solid cancer known to be associated with better prognosis in febrile neutropenic patients compared to other hematologic malignancies [25]. However, lung cancer patients might have smoking and age-related comorbidities such as chronic obstructive pulmonary disease and interstitial lung diseases in which *Pseudomonas aeruginosa* is frequently present. Old age is associated with high mortality in lung cancer patients with febrile neutropenia, although the risk of lung cancer is low in neutropenic patients [26].

In our study, smoking was a common risk factor for pneumonia development in total lung cancer patients and patients who received treatment that included cytotoxic chemotherapy. Smoking is known to be associated with lower-socioeconomic status, poor diet, increased alcohol consumption, and reduced physical activity. The relationship between smoking and community acquired pneumonia has been reported [27]. Smoking affects the loss of cilia, inhibits alveolar macrophage function, mucous gland hypertrophy, and increases goblet cells. This causes microbes to be present and widespread in the bronchus. Oxidative stress and cytokine release are triggered, leading to immune response. This makes the bronchial mucosal epithelium more inflammatory and susceptible to infection [28]. Nicotine can suppress natural killer (NK) cell activity. NK cells are usually activated immune response against viral infections [29–31]. Agostini et al. [16] have noted that smoking increases postoperative pulmonary complications such as increased hospital mortality, intensive care unit admission rate, and hospital length of stay. Jung et al. [32] have also shown that smoking is one of independent risk factors for the development of post-operative pneumonia in cancer patients. Smoking cessation before surgery can reduce postoperative complications [33]. The difference in pneumonia occurrence between current smokers and ex-smokers was not apparent in our study. Further studies are needed to clarify effects of smoking cessation duration before treatment in lung cancer patients.

In advanced stage cancer patients, infection occurs more easily due to bronchial permeability disorders as pressure on the bronchial walls increases caused by enlargement of tumor mass or lymph nodes [21]. In addition, cancer metastasis to the bone marrow can cause leukopenia and anemia [34]. Moreover, cough reflex impairment might be triggered by narcotics, psychotropic therapy, or metastases to the brain [35]. Cancer related treatments can cause pneumonia development in patients with advanced staged lung cancer. Most drugs used for chemotherapy suppress the function of the immune system. Alkalizing drugs, antimetabolites of purines, pyrimidines, and folic acid all have immunosuppressive effect [36]. In our study, advanced staging was associated with pneumonia development in lung cancer patients.

There was a difference in the occurrence of pneumonia according to the pathologic type. Squamous cell carcinomas are much more commonly endobronchial lesions protruding into and obstructing large central bronchus, while adenocarcinomas are much more commonly peripheral [37]. Thus, post-obstructive pneumonia occurs frequently in squamous cell carcinoma. SCLC is strongly associated with smoking. Most SCLC patients receive treatment based on cytotoxic chemotherapy and/or radiotherapy. They are vulnerable to infections due to suppressed cellular immunity [38].

This study has some limitations. First, it was a retrospective study. Nonetheless, our study included patients from multiple centers with a large sample size. Second, our study did not investigate whether pneumococcal vaccine (PSV 23 or PCV 13) was given. Chiou et al. [39] noted that the cumulative hospitalization rate for pneumonia over 2 years in lung cancer patients was 37.1% in the vaccinated group and 55.4% in the non-vaccinated group. Third, exclusion of radiation pneumonitis was not complete because differential diagnosis between pneumonia and radiation pneumonia was based on retrospective medical chart and radiologic findings reviews. However, we tried to exclude cases suggesting radiation pneumonitis such as those with relatively sharp demarcation consolidation or GGO with limited radiation field as much as possible.

Despite these limitations, the present study had several strengths. This study was a 7 multi-center study with a large sample size. To identify the risk factors for pneumonia development in patients with lung cancer, pneumonia patients were compared with a relatively large number of non-pneumonia patients. In addition, when the patients were newly diagnosed with lung cancer, data was collected prospectively after registration and the bias was minimized.

## Conclusion

In conclusion, old age, smoking, and neutropenia were common risk factors for pneumonia development in total patients with lung cancer and patients who received treatment that included chemotherapy. Thus, cautious monitoring of elderly patients, especially those who receive cytotoxic chemotherapy or have a history of smoking, is important. Future studies are needed to clarify effects of smoking cessation before treatment on pneumonia development in lung cancer patients.

## Data Availability

Data analyzed in the current study are not publicly available. They may be made available from the corresponding authors upon reasonable request.

## Abbreviations

TKI: Tyrosine kinase inhibitor
CMC: Catholic Medical Center
BMI: Body mass index
SCLC: Small cell lung cancer
SBRT: Stereotactic body radiation therapy
CCRT: Concurrent chemoradiation therapy
GGO: Ground glass opacity
HRs: Hazard ratios
CIs: Confidence intervals
OS: Overall survival
NK: Natural killer
PSV: Pneumococcal polysaccharide vaccine
PCV: Pneumococcal conjugate vaccine
